# Secure Indoor Localization Based on Extracting Trusted Fingerprint

**DOI:** 10.3390/s18020469

**Published:** 2018-02-05

**Authors:** Juan Luo, Xixi Yin, Yanliu Zheng, Chun Wang

**Affiliations:** School of Information Science and Engineering, Hunan University, Changsha 410012, China; yinxixi@hnu.edu.cn (X.Y.); yanliu_zheng@hnu.edu.cn (Y.Z.); cwang@hnu.edu.cn (C.W.)

**Keywords:** fingerprint, indoor localization, secure, trusted factor, WiFi

## Abstract

Indoor localization based on WiFi has attracted a lot of research effort because of the widespread application of WiFi. Fingerprinting techniques have received much attention due to their simplicity and compatibility with existing hardware. However, existing fingerprinting localization algorithms may not resist abnormal received signal strength indication (RSSI), such as unexpected environmental changes, impaired access points (APs) or the introduction of new APs. Traditional fingerprinting algorithms do not consider the problem of new APs and impaired APs in the environment when using RSSI. In this paper, we propose a secure fingerprinting localization (SFL) method that is robust to variable environments, impaired APs and the introduction of new APs. In the offline phase, a voting mechanism and a fingerprint database update method are proposed. We use the mutual cooperation between reference anchor nodes to update the fingerprint database, which can reduce the interference caused by the user measurement data. We analyze the standard deviation of RSSI, mobilize the reference points in the database to vote on APs and then calculate the trust factors of APs based on the voting results. In the online phase, we first make a judgment about the new APs and the broken APs, then extract the secure fingerprints according to the trusted factors of APs and obtain the localization results by using the trusted fingerprints. In the experiment section, we demonstrate the proposed method and find that the proposed strategy can resist abnormal RSSI and can improve the localization accuracy effectively compared with the existing fingerprinting localization algorithms.

## 1. Introduction

In recent years, with the development of wireless technology, the demand for location-based services (LBS) is increasing [1]. As we all know, tracking, localization and navigation are hot topics in academia and industry [2]. In addition, the popularity of mobile computing devices inside buildings promotes the development of indoor localization. An accurate indoor location service gives people better convenience, especially in hospitals [3], airports, car parks, underwater [4], mines, and so on. Therefore, in the future generation of communications networks, accurate, reliable and real-time indoor localization and position-based protocols and services are required.

Nowadays, a variety of indoor localization technologies have been derived, including GPS, infrared, ultrasound (US), time of flight (TOF) and magnetic (MG). Although the Global Navigation Satellite Systems (GPS, GLONASS, Galileo or Beidou) support localization, they cannot operate satisfactorily in indoor scenarios due to numerous factors [5]. Many different localization techniques and indoor localization systems (IPS) have been proposed to deal with indoor localization [6]. A few efforts have been done in the context of infrared (IR) wireless networks. The limited range of IR networks is a handicap in developing this technology. Furthermore, the IR network is often deployed for the purpose of locating people and does not provide traditional data networking service [7]. ToF mainly estimates the distance by measuring the signal transmission time between nodes. However, this technique has some drawbacks. The transmission of an ultra-wide band signal requires relatively large energy consumption, and the effective measurement distance is short. The distance measured by ultrasonic technology can reach the precision of centimeters. Ultrasonic technology can make the structure of the system relatively simple, and the light does not interfere with the ultrasonic. The disadvantage is that the ultrasonic signal attenuates in the air, and the small ultrasonic probe can only reach the meter level. If the reflection range is adopted, it will also be affected by the multi-path effect, and the ultrasonic wave is the method of line-of-sight propagation, so it is not suitable for large-scale occasions. In addition, the transmitting nodes with ultrasonic sensors usually need to continuously broadcast the acoustic signal, which is a significant expense for the whole network, which increases the cost of the hardware facilities. The tracking and locating of equipment based on the principle of MG [8] is characterized by its portability, simple operation and low cost. However, the system also has many shortcomings as follows: The MG transmitting device needs to continuously emit MG signals with high power consumption and high requirements on the power supply system. In the tracking and positioning, the technology is seriously disrupted by the external MG interference; the operation is cumbersome; the error is large; and the requirements of the monitoring personnel are high. Technologies based on existing infrastructure include WiFi [9], ZigBee, Bluetooth, UWB and RFID [10]. To estimate the position, we need to measure some signal characteristics. Common signal features include arrival time (TOA), arrival time difference (TDOA), receiving signal strength indication (RSSI), etc. [11].

Among many RSSI-based indoor positioning technologies, the more popular positioning technology is based on WiFi wireless signals since the IEEE802.11 APs were deployed as wireless local area networks (WLANs). Prior to the widespread adoption of WiFi technology, radio frequency (RF)-based [7] RADAR systems have been proposed that determine a user’s location by recording and processing signal strength information from multiple base stations. In the meantime, some researchers have proposed hybrid indoor positioning methods such as using 2D markers to supplement WiFi intensity or using different wireless technologies such as cellular GSM, DVB, FM and WLAN to locate users. However, network access points with higher radio coverage, such as GSM and FM base stations, contribute less to indoor positioning. Some researchers have tried to build indoor positioning systems such as RFID and ZigBee in combination with other low-power technologies. However, these technologies require a large number of low-power devices to support the positioning process. Some researchers are also working on multi-source data combination positioning technology, which can improve the positioning accuracy, but the system cost will be greatly increased. WiFi is very popular and is one of the most commonly-used wireless technologies in the room. It is often used for wireless Internet connections in many buildings, such as shopping malls, factories and other public places. One great advantage of this technology is that it is widely used in a variety of devices, such as mobile phones, laptops and tablets, which users usually use [12].

WiFi based on indoor location systems typically uses WiFi access points (APs) to calculate the user’s possible locations [13]. In the indoor environment, the extensive application of WiFi makes WiFi-based IPS possible in many places. The localization method based on RSSI [14] has become the mainstream method of indoor localization, with the advantages of low cost, wide coverage and no need for any other hardware [15]. The method is generally divided into two categories. The trilateration algorithm is based on the principle of distance crossover; the fingerprint location method is based on the database and the specific geometric or probabilistic algorithm to calculate the location of unknown points [16]. The extraction of RSSI is crucial for trilateration algorithms and fingerprinting methods [17]. The refinement of RSSI directly affects the accuracy of positioning; Bisio; et al. [18] consider that different handsets can have an impact on RSSI, and they propose a localization method over different smartphones. It eliminates the impact of different handsets on RSSI.

WiFi fingerprinting [19] is based on the RSSI associated with each wireless access point (APs) and is compared to a fingerprint database [20]. This fingerprint database contains a set of previously-recorded fingerprints at known reference points. The location of the target is usually determined by calculating the distance or fingerprints collected by the device and the fingerprints contained in the reference database [21]. However, there is an important disadvantage that the WiFi signals are affected by a number of factors, including changes in personnel activities and environmental facilities. Moreover, the hardware properties of APs also have a serious impact on RSSI and affect the performance of an indoor localization system. At present, some researchers have begun to discuss how to select an appropriate AP for positioning. Chen; et al. [22] discusses a method of selecting APs for indoor localization. An important goal of indoor location estimation systems is to increase the estimation accuracy while reducing the power consumption. Choosing a suitable AP can reduce the power consumption of the AP and, at the same time, achieve a better positioning effect.

For example, in underground mines, the environment is complicated and changeable, and when it changes, it will lead to the mutation of RSSI [23]. Moreover, considering the construction conditions of underground mines or unforeseen emergencies, there are potential safety hazards in the physical equipment in the location environment. When an AP is impaired, the RSSI of the AP loses the original transmission characteristics. Larger localization errors caused by these disturbances are more serious, especially for emergency rescue and target tracking in the current situation, which will lead to the target not being able to be accurately located.

**Figure 1:**
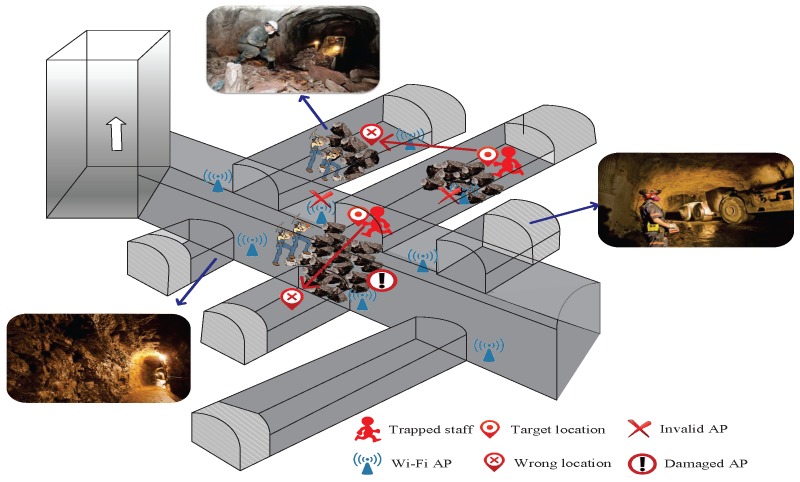
Problem scenario.

Figure 1 depicts the scenario of using impaired APs to locate when environmental changes occur. The impaired APs may be caused by plenty of factors, including earthquakes, explosions or just regular maintenance. In this paper, we describe the localization problem when there are impaired APs by using a mine collapse scenario. As shown in Figure 1, when the environment changes, APs are impaired and do not work properly, and if you cannot identify impaired APs in time, it will continue to use the RSSI of the impaired APs to locate. The RSSI of the impaired APs will be mutated, making it poorly matched with the fingerprint database and resulting in incorrect location information. In the case of emergency rescue, giving the wrong target location will mislead the rescue work and miss the best time to rescue.

In view of the above problems, this paper analyzes the known WiFi fingerprinting methods for indoor localization. We examined various aspects of WiFi fingerprinting, including impaired APs and changes of the localization environment. We analyzed the RSSI characteristics of impaired APs. A method of identifying impaired APs is found by analyzing and comparing the RSSI, which is affected by the environment and the impaired APs. In the position estimation algorithm, the impaired APs are filtered, to ensure the fingerprints are safe and reliable in the position estimation.

The rest of this article is organized as follows. Section 2 reviews the related work on reducing localization errors. Section 3 analyzes the characteristics of RSSI and the problems caused by different influences. Section 4 introduces the research content proposed in this paper. Section 5 shows the experimental analysis and the experimental results of the proposed work. Section 6 gives the conclusions drawn from this work.

## 2. Related Work

Understanding RSSI data analysis is crucial to indoor localization algorithms. Gaussian or log-normal distributions are currently used to simulate the RSSI randomness. Large-scale measurements [24] show that the RSSI histogram fits the Gaussian distribution. In addition, smartphones are smaller and have less computing power than laptops, so smartphones are more sensitive to environmental changes when collecting RSSI for indoor location applications. This stimulated our analysis and research on RSSI and improved the indoor localization accuracy of smartphones.

A basic requirement of fingerprint recognition is that the fingerprint must be accurate and updated in time. Hao Jing et al. [25] proposed a new fingerprinting training method, which can set the fingerprint database when the user enters an environment without a prior database. Previous databases can also be updated by collecting information from surrounding users. Both historical data and new data have been applied to update the database, and reduce training costs. The authors in [26,27] studied autonomous crowdsourcing methods to train and update WiFi databases. For crowdsourcing databases, the accuracy of the fingerprint database is crucial to estimating locations. In [28], the authors improved fingerprinting performance by allowing users to interact with the system. However, these methods require active collaboration with users who may not be willing or likely to make mistakes.

Many scholars have done much research on the factors that affect the position of fingerprints. Wallbaum [29] used several analytical models and empirical multi-wall radio propagation models to study the impact of the following key parameters on indoor location: RSSI bias, number of access points and granularity of the grid. Krishnakumar and Krishnan [30] made some important observations about the estimation uncertainty and the dependence of various factors. The observed factors include the signal variance, the number of APs and the distance between APs and signal propagation constants. Dempster et al. [31] introduced RSSI analysis of variance, indicating that different users’ orientations at the reference point affect the RSSI captured by the device. The result is that the relationship between the true distance of two fingerprints and the RSSI distance is poor. One of the reasons for the localization error is comparing fingerprints with imperfectly suitable reference fingerprints.

In order to localize in the underground environment, Liu [32] and others proposed a target method of underground localization based on distance constraints. RSSI technology is used to measure distance, but the radial error is large because the algorithm contains approximation. In [33], in order to improve the localization accuracy of wireless sensor networks in underground tunnels, an anchor node selection mechanism was proposed. The mechanism is based on the tunnel’s three-dimensional spatial characteristics and the choice of wireless communication frequency: firstly, sorting from high to low based on RSSI values; secondly, selecting four anchor nodes with higher RSSI by Gaussian elimination. The algorithm selects the anchor nodes according to the RSSI level, but when there are fewer anchor nodes, the unstable anchor nodes may still be selected.

Haeberlen et al. [34] studied the time-varying phenomenon of RSSI. The authors point out that environmental impacts, such as interference and movement of people or mobile nodes, cause time-dependent fluctuations in RSSI and severely reduce the performance of the localization system. A discussion of sample correlations was proposed in [35], and the authors used these highly correlated RSSI samples in their localization system. However, in the RSSI time series, there are no detailed reports to study the stationarity or correlation of time-varying phenomena. In addition, over time, signals from APs may disappear due to the intermittent degradation of the radio channel. This can result in null values of the RSSI in position determination and can lead to negative effects on performance, computational complexity and indoor localization system scalability [36].

This paper presents a secure indoor location algorithm based on the trusted location fingerprint. The proposed algorithm can be applied to many scenarios, including earthquakes, explosions and floods. The algorithm can identify the impaired AP after an accident and select the safe and trusted AP to locate. For example, in the case of a magnitude 5.0 earthquake, it may lead to the collapse of a few tunnels and the breakdown of some pipes. In this case, the algorithm can exclude the impaired APs and select safe APs to locate the target and carry out rescue work. In the event of a major accident, the algorithm identifies that all the APs have been impaired, and then, the positioning system may fail. If the positioning system can operate, the reference value of the positioning result will be determined according to the number of impaired APs. The main contributions of this paper are as follows:

A fingerprint database update model based on error analysis is introduced. According to the characteristics’ analysis of RSSI and error verification, the fingerprint database is updated partially. It can reduce the update overhead and avoid the introduction of unreliable fingerprints when updating the fingerprint database.
We propose a trust factor extraction mechanism based on the voting algorithm. APs are voted through the test of RSSI based on reference points. According to the vote, results extract APs’ trust factor, reducing the impact of untrusted APs on localization.
We designed a fingerprint matching algorithm based on weight distribution. By comparing the RSSI value received on the online phase with the offline phase, we can filter out the broken APs and the new APs. The trust factor of APs is used in the calculation of fingerprint weight, to reduce the impact of untrusted fingerprints on the location algorithm.

## 3. Localization Model

**Figure 2:**
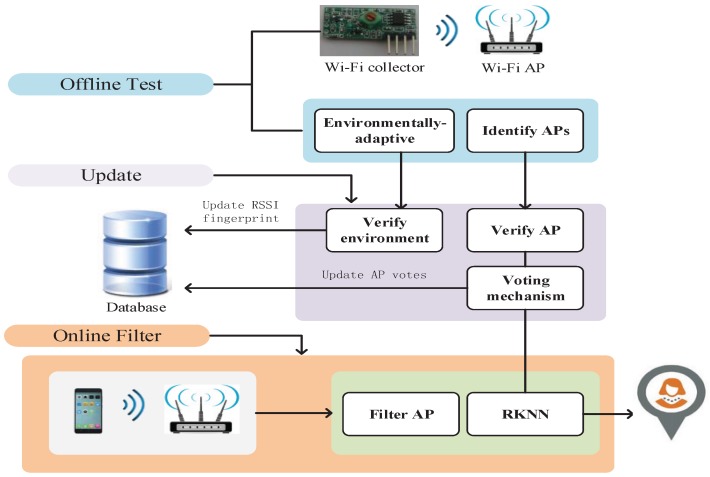
Reference system architecture.

WiFi fingerprinting is a well-known indoor localization solution, which relies on a fundamental assumption: the WiFi signals measured in the environment have a unique signature that comprises the WiFi fingerprint at a given position. In this section, we only analyze the changing characteristics of RSSI under different interferences. According to the experiment and scene analysis, this paper presents a fingerprint extraction mechanism. The system framework is shown in Figure 2. The symbols used in this paper are shown in Table 1.

The algorithm proposed in this article is to detect the secure status of the positioning device. In positioning, if the localization device is damaged or fails, the proposed algorithm filters the device by detecting the secure status of the localization device and selects the data of the localization device that is safe and trustworthy to locate the target. Therefore, when emergencies such as earthquakes, floods and explosions occur in the positioning environment, the proposed algorithm can identify the secure physical devices and locate the targets with safe and reliable data. This paper improves the robustness of the positioning system by using the security identification algorithm.

### 3.1. Fingerprinting Localization

The fingerprint-based localization process is usually divided into two stages: the offline sampling phase and online localization phase. The offline phase creates a fingerprint database. The specific content collects the RSSI at all reference points in the locating area. The RSSI of APs at a reference point are taken as the fingerprint. All the fingerprints of the reference points form a fingerprint database. Each piece of fingerprint information corresponds to the position information. The indoor localization environment is variable, so in order to reduce the interference of environmental factors, we continuously sample at each sampling point and calculate the average. According to the RSSI of the location to be positioned, the fingerprint matching algorithm is used to calculate the similarity degree and obtain fingerprints that have high similarity. Then, the reference position coordinates corresponding to the fingerprint are taken as the actual position of the user. The original fingerprint localization algorithm keeps the offline fingerprint database static, but for the variable environment, if the fingerprints in the database remain unchanged, the final localization results will have greater deviation.

Fingerprint-based localization technology not only should consider the differences of the fingerprint between the online phase and offline phase, but also needs to consider the AP’s physical security issues. If the AP is impaired due to external conditions and at the same time the AP is still used for localization, the localization accuracy will be affected by the AP. In addition, if there are new APs in the environment and the fingerprint of the APs is not updated in the fingerprint database, the localization error may also be increased if the APs are used for localization.

### 3.2. Error Sources of RSSI

Nowadays, most researchers usually consider the loss problem of RSSI propagation, and they take corresponding measures to deal with the problem. However, when performing localization in harsh indoor environments such as in underground coal mine localization, the safety of staff needs to have attention paid to it; at the same time, the safety of physical equipment should also be of concern to ensure that the system that uses the equipment can work normally. When the APs are impaired, the RSSI will be changed. In the fingerprint-based localization system, if there are wrong RSSI, this will cause a large localization error. In order to make the localization result more accurate, we need to identify the environmental factors and other external conditions and select more stable and trusted fingerprints for localization.

This section analyzes the impact of APs themselves on localization accuracy. In the underground coal mine localization environment, due to the variability of the environment and the complexity of the tools used, the physical equipment may be impaired to varying degrees. The impaired extent of the equipment can be roughly divided into: slightly impaired and completely destroyed. Slightly impaired represents that the device can transmit signals, but due to the equipment being impaired, the signal transmitted by the equipment is weak or unstable. Completely destroyed means that the equipment is unable to work, so it cannot transmit signals. In order to avoid the impact of impaired equipment on the localization accuracy, we need to identify different degrees of impairedness and then apply different solutions for different situations. For slightly impaired APs, we reduce the trust value of the APs in localization so that the fingerprint in the fingerprint database can be more compatible with the fingerprints in the online phase. The deployment and the number of APs also affect the localization result. In order to reduce equipment overhead, when deploying APs, we should achieve the best effect with minimum APs. Therefore, if there are destroyed APs in the localization environment, we should identify and handle the APs in time so as to avoid the impact of the APs on the localization system.

**Figure 3:**
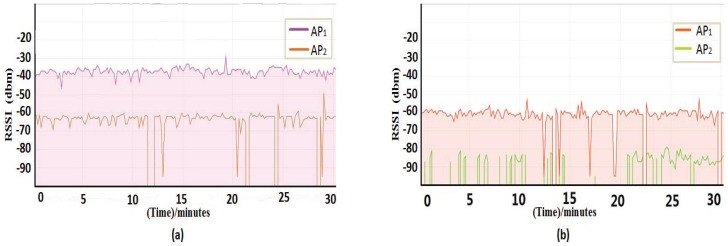
Signal strength analysis. (**a**) Description of RSSI at 2 m away from the AP. (**b**) Description of RSSI at 6 m away from the AP.

We carry out an experimental analysis of the RSSI when there is an impaired AP. First, we fixed the distance between the measuring location and the AP, then we collected the RSSI for impaired AP and normal AP, respectively. In order to verify whether the distance between the measuring location and the AP may have an effect on the distinction between normal AP and impaired AP, we increase the distance between the AP and the measuring location, then collect the RSSI values when the AP is impaired and the AP is normal. The RSSI was recorded every five seconds, and the total duration was 20 min. The experimental results are shown in Figure 3.

It can be seen from Figure 3 that the RSSI of the impaired AP is obviously lower than the RSSI of the normal AP, no matter the distance between the measuring location and the AP. Furthermore, the RSSI of an impaired AP is extremely unstable compared with the RSSI under normal conditions. If the AP is completely destroyed, it is impossible to receive the AP’s RSSI no matter how close we are to the AP.

**Figure 4:**
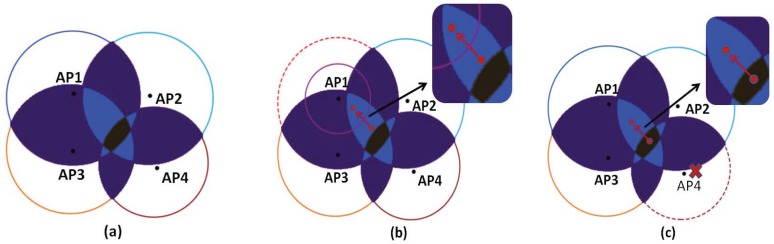
Localization error analysis. (**a**) Description of the normal position area. (**b**) Description of the position area when an AP is impaired. (**c**) Description of the position area when an AP is broken.

Figure 4 shows the problems with the localization system when an AP is impaired. Figure 4a shows the target location area that the localization system obtains based on the RSSI under normal conditions. When an AP is slightly impaired, the RSSI will decrease. If we do not know that the AP is impaired and use it for localization, the localization result will be affected by the AP, resulting in a large error in the localization result. Figure 4b shows that when an AP is impaired, the localization system obtains the change of the target location area compared with the normal situation. When an AP is impaired, the RSSI will decrease, so the coverage area of the AP will be smaller, and its RSSI is not stable. If the AP is used for localization, its localization interval will be affected by the AP so that the localization area moves toward the wrong area, resulting in a larger localization error. Figure 4c shows the change of the localization area when an AP is destroyed. If it is still used for localization when the AP is destroyed, the obtained localization interval will be offset and become larger, and the localization accuracy will be reduced. Therefore, we need to identify the impaired AP to ensure that the location fingerprints in the localization system are safe and trusted.

Based on the above analysis, the impaired AP has a serious impact on localization accuracy. Therefore, the localization system needs to be clear about the security status of APs, then perform the appropriate treatment. According to the analysis of the change of RSSI in Figure 3, we can see that the three states of normal, impaired and destroyed APs can be distinguished according to the change of RSSI and the fluctuation degree.

### 3.3. Effective Screening Model

In the underground coal mine localization environment, the length and width of the coal mine, the number of mines and the position of obstacles are dynamically changing. The size of the RSSI will change in real time due to the environmental factors. The traditional static fingerprint database localization technology is not suitable for the scene whose environment changes frequently. In order to adapt to the variable environment of coal mines, we deploy some reference anchor nodes of known positions in the localization environment and update the fingerprint database according to the localization results of the anchor nodes. While updating the fingerprint database, this paper analyzes and compares the changes of RSSI received by reference anchor nodes to identify impaired APs.

**Figure 5:**
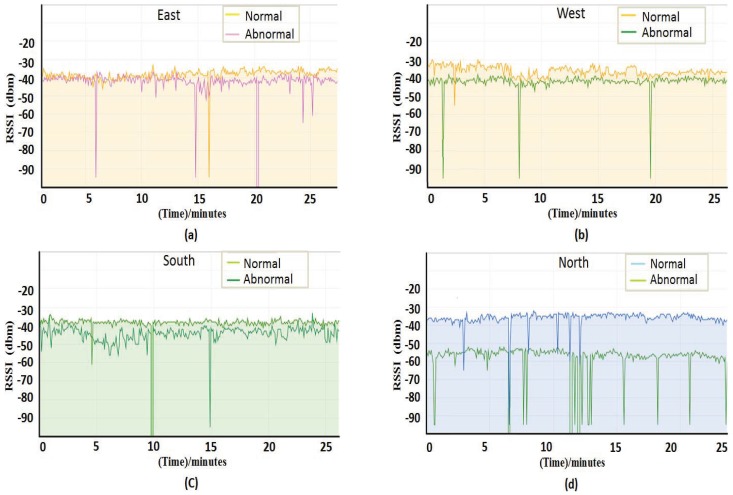
Environmental impact on RSSI. (**a**) RSSI values that are measured in the east. (**b**) RSSI values that are measured in the west. (**c**) RSSI values that are measured in the south. (**d**) RSSI values that are measured in the north.

In the underground coal mine localization environment, the physical equipment and the personnel concentration areas are variable, so there are some difficulties in distinguishing the impaired AP from the environment-affected APs. In order to choose a better filter mechanism, we analyzed the characteristics of RSSI, which is affected by the environment. We collected RSSI of environment-affected APs and normal APs in four different directions as shown in Figure 5. The four images in Figure 5 represent RSSI obtained in four different directions, each of which represents normal RSSI and disturbed RSSI. It can be seen from Figure 5 that the impact of environmental factors is regional, which is that only the areas affected by the environment have a change of RSSI, while RSSI in other regions are not affected. From Figure 5, we can draw the conclusion that the obvious change of RSSI can only occur in the direction of environmental impact, whose change do not affect all the reference anchor points.

**Figure 6:**
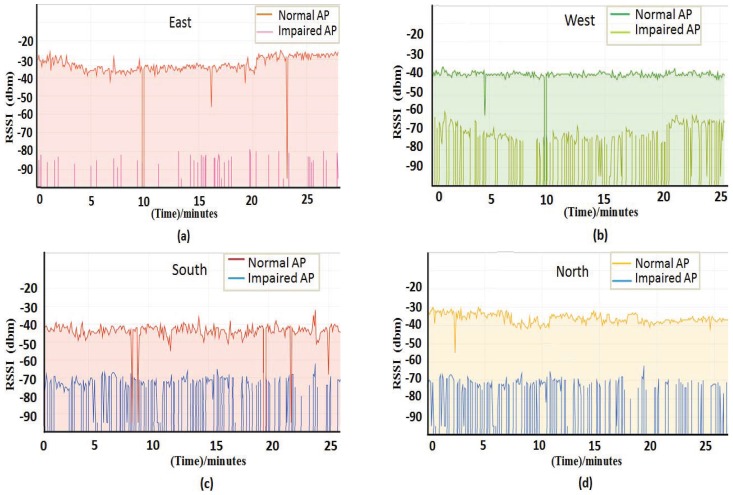
Impact of impaired AP on RSSI. (**a**) RSSI that are measured in the east. (**b**) RSSI that are measured in the west. (**c**) RSSI that are measured in the south. (**d**) RSSI that are measured in the north.

In order to find the difference between the impaired AP and the environment-affected AP, we measured the RSSI of the AP in four different directions when the AP was impaired as shown in Figure 6. Figure 6 shows the comparison of RSSI regarding impaired AP and normal AP in four different directions. As you can see from Figure 6, the RSSI around the AP change when the AP is impaired; the surrounding reference anchor nodes can detect the change of the RSSI and can obtain the fluctuation degree according to the RSSI collected over a period of time. However, when the environment changes, only the reference anchor nodes in a certain direction may detect the change of the fluctuation degree and the size of RSSI. Therefore, the method of voting by reference anchor nodes can be used to identify the impaired AP.

Moreover, as can be seen from Figure 6, the RSSI fluctuation value of the normal AP is small, and the fluctuating RSSI value of the impaired AP is large. Furthermore, the normal AP’s RSS value is greater than the value of the impaired AP. Due to the existence of RSS heterogeneity, such as RSS offset, there may be some error if comparing the average value of RSS before and after updating the database to determine whether the AP is normal. In addition, using the change of standard deviation before and after the update database as a voting standard, we can screen out APs with relatively stable RSSI values. Using these APs for positioning can make the positioning result more accurate. Therefore, this paper compares the standard deviation of RSSI before and after updating the database to vote on the impaired AP.

When an AP is impaired, if the reference anchor node around it votes on the AP using the voting mechanism, the number of votes of the impaired AP will increase. If the weight of AP is given according to the number of votes, the trust value of the AP fingerprint will be obtained when localization has occurred, and the impact of impaired AP on the localization result will be reduced. If the AP is affected by environmental factors, only the reference anchor node in a certain direction will vote on the AP and will not cause the AP to have a higher number of votes. Therefore, the AP still has a higher trust value. If the surrounding environment of an AP is changed greatly and the fingerprint fluctuation is large, the votes of the AP will be very high. Therefore, using this method one can also filter APs that are greatly affected by the unstable environment. In this way, the impaired APs can be well identified, filtering the untrusted fingerprint of impaired APs. If none of the reference anchor nodes around an AP can receive the RSSI, it can be concluded that the AP is broken; the fingerprint of the AP in the fingerprint database is updated; and the AP is filtered during localization.

## 4. Secure WiFi Fingerprint Localization

The process of the indoor safety localization algorithm based on trusted fingerprint is divided into three steps: The first is the offline testing phase, in which an offline fingerprint database is established, the location of reference anchors nodes is calculated and the RSSI of APs are compared according to localization errors to detect whether the environment or impaired APs have an influence. The second is the offline update phase, in which the location of the reference anchor node is calculated again using the fingerprint after detection to verify whether there is any impact from the environment or the impaired APs. According to the verification result, the APs are voted on, the weight of APs are obtained, then the fingerprint and weight of the APs are updated. The third stage is the online filtering phase. In this stage, the impaired AP is filtered by the RKNN (reliable-KNN) algorithm. The RKNN algorithm calculates the weight of each reference position according to the similarity of reliable fingerprints and selects the K positions with the highest weight to calculate the weighted average value, which is the final positioning result. Furthermore, the destroyed APs and new APs are filtered by comparing the number and type of APs in the offline and online phases.

### 4.1. Offline Testing Phase

#### 4.1.1. Collect Offline RSSI

In order to build an offline fingerprint database, we need to collect RSSI in the localization area. We divide the indoor network coverage area into N=n×n square grids; *N* is the total number of meshes; and the grid is encoded in coordinates. We consider each grid as a reference point. The offline fingerprint database is a set of all reference point fingerprints. For the fingerprint-based localization algorithm, the larger the grid, the greater the positioning error, and the smaller the grid, the greater the workload of establishing a fingerprint database. We set the communication radius of an AP as *r*, when the range of the number of meshes $N_{grid}$ is $\left(2S_{region}/r^{2}\right)\leq N_{grid}\leq \left[S_{region}/\left(10^{(\mathrm{lg}\left(I_{\min}\right)-\wedge )/\alpha }\right)\right]$, and the acquisition cost of the offline stage is low [17]. When collecting fingerprints, we set the size of the grid to 0.5 m × 0.5 m.

As for the complex environments, we collect multiple samples and get their average. In order to calculate the RSSI’s average of $AP_{i}$ received at grid *j*, we place a reference anchor node at grid *j* to receive the RSSI sent by $AP_{i}$. Assuming that the total number of RSSI samples received by the reference anchor node is *q*, then the average RSSI received by the reference anchor node placed in grid *j* can be calculated as follows:

(1) $$\varphi_{i,j}=\overset{q}{\underset{\tau =1}{\sum }}\varphi_{i,j}\left(\tau \right)/q.$$

where $\varphi_{i,j}\left(\tau \right)$ is the τ-th RSSI of the grid *j* received. $\varphi_{i,j}$ is the average RSSI of the grid *j* received. The number of APs is L. The initial database matrix Ψ is as follows:

(2) $$\Psi =\left\{\begin{array}{cccc} \varphi_{1,1} & \varphi_{1,2} & \cdots & \varphi_{1,N}\\ \varphi_{2,1} & \varphi_{2,2} & \cdots & \varphi_{2,N}\\ \vdots & \vdots & \ddots & \vdots \\ \varphi_{L,1} & \varphi_{L,2} & \cdots & \varphi_{L,N} \end{array}\right\}.$$

If the grid *j* is outside the communication range *r* of $AP_{i}$, that is the reference anchor node placed in grid *j* cannot receive the RSSI from $AP_{i}$, then let $\varphi_{i,j}=0$.

#### 4.1.2. Construct the Standard Deviation

Compared with the traditional fingerprint localization method, the algorithm in this paper records the standard deviation of the RSSI of each AP in the fingerprint database, according to the size and standard deviation of RSSI voting rules. When the fingerprint database is updated, we need to determine whether the fingerprint in the fluctuations is in the normal range; if so, the fingerprint is trusted; otherwise, it is not trusted. In this way, we can obtain the trusted value of APs and determine the contribution value of APs according to the trust value in the localization process, reducing the localization error caused by the abnormal RSSI of APs.

Now that the total number of grids is *N*, the standard deviation of $AP_{i}$ at the *j*-th grid is $b_{i,j}$, and the RSSI standard deviation matrix *B* is as follows:

(3) $$B=\left\{\begin{array}{cccc} b_{1,1} & b_{1,2} & \cdots & b_{1,N}\\ b_{2,1} & b_{2,2} & \cdots & b_{2,N}\\ \vdots & \vdots & \ddots & \vdots \\ b_{L,1} & b_{L,2} & \cdots & b_{L,N} \end{array}\right\}.$$

where $b_{i,j}=\sqrt{\overset{q}{\underset{\tau =1}{\sum }}{\left(\varphi_{i,j}\left(\tau \right)-\overset{q}{\underset{\tau =1}{\sum }}\varphi_{i,j}\left(\tau \right)/q\;\right)}^{2}/q\;},i=1,2,\ldots ,L,j=1,2,\ldots ,N$ is the degree of volatility of the RSSI from $AP_{i}$ at the *j*-th grid. *q* is the number of RSSI samples received by the reference anchor node. *L* is the number of APs.

#### 4.1.3. Build the Offline Database

The offline fingerprint database includes the coordinate of each anchor node, the RSSI of each reference point in the offline phase, the standard deviation of the RSSI of each AP, the distance between the grids and the vote matrix *V*. The initial value of vote matrix is zero. As shown in Equation (4):

(4) $$\left(X,Y;x,y;\Psi ;B;D;V\right),$$

where $\left(X,Y\right)$ is the coordinate of the AP and $\left(x,y\right)$ is the grid coordinate. At the same time, we need to establish the distance matrix between the grids to update the fingerprint database.

### 4.2. Offline Update Phase

Due to the online phase and the offline phase having a certain time difference, during which the AP is likely to be impaired, resulting in the RSSI being biased against the existing RSSI in the database, if we do not detect the trusted value of APs, the impaired AP will have a greater impact on the localization results. Therefore, in order to determine the credibility of APs, the algorithm proposed in this paper votes for APs, then updates the fingerprint information in the database and the weight information of the APs.

#### 4.2.1. Fingerprint Database Updates

In order to solve the problem of environment changes, we need to update the fingerprint database. We placed some reference anchor points for fingerprint detection in the localization area. The reference anchor nodes are a simple WiFi terminal, which consists of a power supply module, a WiFi transmitter and a receiver module. The function is to periodically receive the RSSI of the surrounding AP the RSSI and location information package sent to the localization server.

Within the communication range of AP, the reference anchor node can obtain the RSSI of APs, record the average of RSSI and the standard deviation of RSSI. Obtain the measurement matrix $\varphi =\left[s_{1},s_{2},\cdots ,s_{L}\right]$ of reference anchor nodes, and then use the RKNN algorithm to estimate the coordinates. The RKNN algorithm is used to calculate the coordinates $\left(x_{i},y_{i}\right)$ of the reference anchor nodes. Because the location of the reference anchor node is known, we can calculate the localization error of the reference anchor node. The error is calculated as follows:

(5) $$\delta_{i}=\sqrt{{\left(x_{0}-x_{i}\right)}^{2}+{\left(y_{0}-y_{i}\right)}^{2}}.$$

where $\left(x_{0},y_{0}\right)$ denotes the actual position of the reference anchor node and $\left(x_{i},y_{i}\right)$ denotes the estimated position. We can decide whether to update the fingerprint database according to the error value $\delta_{i}$. If the localization error exceeds threshold *T*, which is 1 m, we conducted fingerprinting tests and then screened fingerprints. Then, the coordinates $\left(x_{i},y_{i}\right)$ of the reference anchor nodes are calculated again; if the positioning error is reduced, the screened fingerprints and the votes of APs are updated in the database. If the positioning error does not decrease after screening, all the fingerprint information is updated. We obtain the newest RSSI matrix $\Psi^{\prime }$.

(6) $$\Psi^{{}^{\prime }}=\left\{\begin{array}{cccc} {\varphi^{{}^{\prime }}}_{1,s_{1}} & {\varphi^{{}^{\prime }}}_{1,s_{2}} & \cdots & {\varphi^{{}^{\prime }}}_{1,s_{k}}\\ {\varphi^{{}^{\prime }}}_{2,s_{1}} & {\varphi^{{}^{\prime }}}_{2,s_{2}} & \cdots & {\varphi^{{}^{\prime }}}_{2,s_{k}}\\ \vdots & \vdots & \ddots & \vdots \\ {\varphi^{{}^{\prime }}}_{L,s_{1}} & {\varphi^{{}^{\prime }}}_{L,s_{2}} & \cdots & {\varphi^{{}^{\prime }}}_{L,s_{k}} \end{array}\right\}.$$

#### 4.2.2. Voting Mechanism

In order to distinguish the different effects of environmental and impaired AP on RSSI, we conducted an experimental analysis and comparison of RSSI in the presence of environmental impact and impaired AP. As shown in Figure 4 and Figure 5, when there is an environmental impact, the affected range may be in one or both directions; the fluctuation and RSSI may change in this direction. However, when there is an impaired AP, the volatility and average of RSSI received by all the reference points within that AP range will change. There is a clear distinction of the environmental factors and impaired AP on the impact of RSSI. Therefore, the voting mechanism in this article selects the standard deviation as the voting condition, and it can achieve a better filtering effect.

When updating the fingerprint database, the reference anchor nodes measure the RSSI several times. The RSSI standard deviation $B^{{}^{\prime }}$ is recorded according to the multiple measurements.

(7) $$B^{{}^{\prime }}=\left\{\begin{array}{cccc} {b^{{}^{\prime }}}_{1,1} & {b^{{}^{\prime }}}_{1,2} & \cdots & {b^{{}^{\prime }}}_{1,N}\\ {b^{{}^{\prime }}}_{2,1} & {b^{{}^{\prime }}}_{2,2} & \cdots & {b^{{}^{\prime }}}_{2,N}\\ \vdots & \vdots & \ddots & \vdots \\ {b^{{}^{\prime }}}_{L,1} & {b^{{}^{\prime }}}_{L,2} & \cdots & {b^{{}^{\prime }}}_{L,N} \end{array}\right\}.$$

When we update the fingerprint database, we compare the standard deviation before updating. If the standard deviation of the fingerprint that the reference point *j* receives from the fingerprint of $AP_{i}$ is ${b_{ij}}^{{}^{\prime }}$ and ${b_{ij}}^{{}^{\prime }}>b_{ij}+\Delta$, then it is considered that $AP_{i}$ is not trusted, and reference point *j* is used to vote on $AP_{i}$, where Δ is a trust threshold. Finally, we can obtain the vote matrix of each AP for the weight calculation of the APs’ credibility. Each reference point will vote on the APs, if there is an impaired AP. Then, after the end of the algorithm, impaired AP votes are the highest, and the AP that is only affected by the environment has a relatively low number of votes. If an AP is severely affected by the environment, its RSSI is not stable, and it is not suitable for localization. When an AP is seriously affected by the environment, the instability of RSSI will cause the standard deviation to be higher; thus, the number of votes of the AP will be improved. Therefore, the algorithm proposed in this paper can also filter the AP with a serious environmental impact.

We vote on APs based on the size of the standard deviation of the RSSI of the reference anchor node receiving the APs. We calculate the trust degree $W={\left[w_{1},w_{2},\cdots ,w_{L}\right]}^{T}$ of the AP according to the voting result obtained by the voting mechanism, which is expressed as $w_{i}=1-v_{i}/num$; Where $w_{i}$ represents the trust value of AP, $v_{i}$ represents the votes of the ${AP}_{i}$ and num refers to the number of reference points within the AP coverage. In the initial phase, the value of the ticket matrix is zero, and the value of $w_{i}$ is one. The voting mechanism in the algorithm can make the number of impaired AP votes higher and make the weight smaller, so as to achieve the effect of filtering impaired APs. We can also filter out APs that are affected by unstable environmental factors.

Because the APs have been voted on during the update process, the weight of the APs will change. For untrustworthy fingerprints, the weight of the APs will be reduced. In this way, even if there is an untrustworthy fingerprint in the RSSI matrix, its contribution will be reduced due to its lower weight, and the effect on the overall localization result will be reduced.

### 4.3. Online Phase

Because the environment is dynamic and the equipment in the environment has safety hazards, in the offline phase, it may be able to receive the RSSI of APs, and in the online phase, it cannot receive the RSSI of APs; as shown in Table 2. Originally, the location that is closest to the online location is location $\left(x_{1},y_{1}\right)$. However, due to the instability of $AP_{5}$, the RSSI of the $AP_{5}$ is not received by the location to be positioned. If you think that the position to be located is too far from $AP_{5}$ when the fingerprint is matched, it will be matched to the wrong position. As shown in Table 2, if $AP_{5}$ participates in localization, it can be concluded that the position to be located is closest to position $\left(x_{2},y_{2}\right)$. In order to avoid the impact of the problem on the localization results, we need to filter out the AP that has the problem. When we calculate the distance, we only calculate the fingerprint value that is not zero in both the online phase and the offline phase. For a newly-introduced AP, if there is no RSSI information of the new AP in the fingerprint database, you cannot locate using the AP, and this method can filter the newly-introduced APs. The reference anchor node cannot receive the RSSI of broken APs during the online phase, but the RSSI of the APs exist in the database; the broken APs can be filtered by this method, as well.

In the online stage, first obtain the RSSI matrix to be positioned $\varphi =[s_{1},s_{2},\cdots ,s_{n}]$ and then calculate the European distance of the fingerprint to be positioned and the fingerprint at each sampling point; if the position to be located is identified that cannot accept the information from ${\mathrm{AP}}_{j}$, then make $s_{j}=0.$ The distance formula of the φ vector and the RSSI of the reference point in the fingerprint database Ψ are shown as follows:

(8) $$l_{i}=\frac{{\left(\overset{M}{\underset{j=1}{\sum }}w_{j}{\left|s_{j}-\varphi_{i,j}\right|}^{p}\right)}^{1/p}}{M}.$$

where $s_{j}$ is the RSSI of ${\mathrm{AP}}_{j}$ received by the position to be located, $w_{j}$ is the trust value obtained by the number of votes, the *p* is two and *M* represents the number of APs for which the RSSI is not zero in either the online phase or the offline phase. The smaller the value of $l_{i}$, the higher the matching degree of fingerprints. According to this principle, we sort $l_{i}$, in ascending order, and the weight is calculated as follows:

(9) wK−i+1′=li∑j=1Klj

In order to reduce the influence of interference factors on the localization results, the algorithm records the best matching K fingerprint records, obtains the position information of the corresponding reference points and then uses the RKNN algorithm to calculate the coordinates of the position to be located. The equation is as follows:

(10) $$\left(\hat{x}\;,\hat{y}\;\right)=\overset{K}{\underset{i=1}{\sum }}w{{}^{\prime }}_{i}\left(x_{i},y_{i}\right).$$

where $\left(x_{i},y_{i}\right)$ is the position corresponding to the *i*-th reference point in the kreference points and $\left(\hat{x}\;,\hat{y}\;\right)$ is the estimated location of the location to be positioned.

When calculating the euclidean distance, the algorithm adds the weight information of trusted APs. If there are impaired APs in the environment, the false fingerprint information of impaired APs will seriously affect the fingerprint matching. The addition of the AP trust weight can reduce the impact of impaired APs on fingerprint matching and find the fingerprint with higher similarity to the location to be positioned.

## 5. Experiment Analysis

### 5.1. Locating Environment

**Figure 7:**
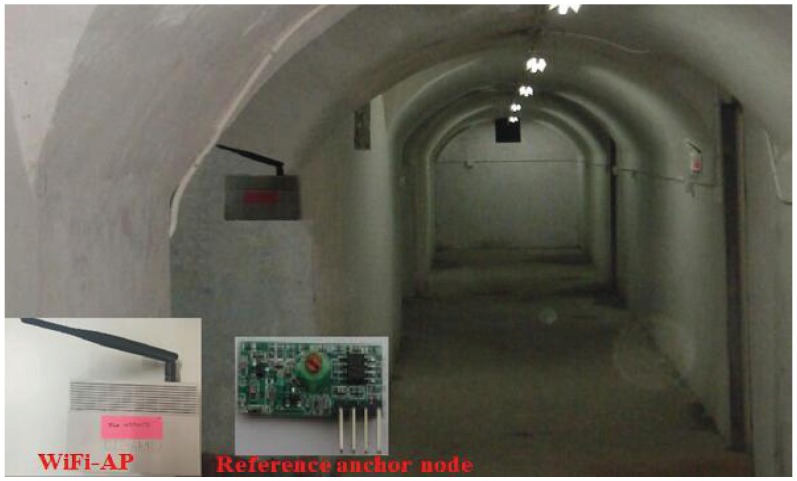
Experimental scene.

We divided the localization area into 400 grids, each grid representing a reference point. The number of APs is 6, and due to irregularities in the experimental environment in the mine, APs are placed irregularly. When deploying an AP, we deploy the APs in an irregularly-shaped form on the premise that each location could receive 6 AP signals. All of the locations were positioned by accepting RSSI from APs. In this paper, experiments were performed in a dynamic indoor environment, and there were impaired APs in the experimental environment. When carrying out the experiment, we collect the RSSI of AP with the mobile phone, establish the fingerprint database and carry out the emulation experiment with the computer. When collecting RSSI fingerprints, we control the acquisition height of 1 m from the ground position, and the AP is placed 2 m away from the ground position. When performing the fingerprint update, the greater the number of times of updating, the larger the energy consumption and the more accurate the positioning accuracy. Because RSSI are highly susceptible to the environment, the frequency of updating the fingerprint database in this article is set to hourly. We choose a bomb shelter as shown in Figure 7. In the experimental environment, we placed 6 APs and 10 reference anchor nodes. The spatial location in the experimental environment was converted into two-dimensional coordinates. Then, we randomly selected a number of locations, used the algorithm proposed in this paper to find the coordinates of the location to be positioned and calculated the localization error. When conducting the experiments, there were people moving around in the lab environment to simulate a dynamically-changing environment. We randomly selected 1 to 2 APs out of 6 APs, removed their antennas and interfered with the power they access, simulating impaired APs.

When there are impaired APs in the environment, in order to fully verify the security performance of the localization algorithm, we carried out the following two types of experiments. The first type of experiment is for the same APs to take different impaired conditions and then observe the resistance of the algorithm when APs are impaired to varying degrees. The second type of experiment is to set up a different number of impaired APs and then observe the resistance of the algorithm when multiple APs are impaired.

### 5.2. The Impact of Trust Threshold on Voting

**Figure 8:**
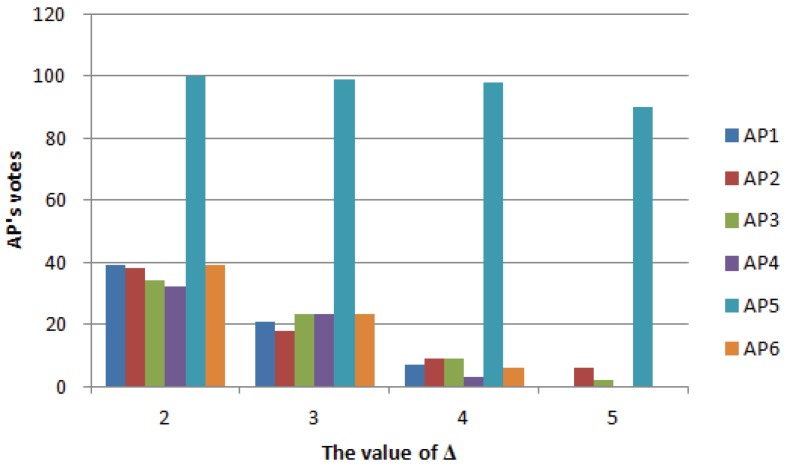
Voting results.

The trust threshold Δ represents the tolerance of the RSSI, which directly affects the voting result of APs. The larger the value of Δ, the greater the change in RSSI that can be tolerated, and the recognition rate for impaired AP and environment-affected APs is lower. Therefore, when the trust threshold Δ is larger, the number of votes for impaired APs and environment-affected APs is lower. In the experiment, the set of environmental interference values is 0 to 10 dbm, the RSSI of the impaired AP decreases by 30 to 50 dbm and the RSSI value of the broken AP is 0. In this experiment, six APs were placed, the impaired AP was $AP_{5}$ and the rest of APs were disturbed by the normal environment. Figure 8 shows the result of this algorithm voting when the AP is impaired. In the experiment, we convert the number of votes for each AP into percentiles, that is the maximum number of votes is 100. If there are large metal objects (e.g., coal trams) in the environment that would generate large disturbances in WiFi signals, according to the voting mechanism, the APs will get a higher number of votes. The smaller the weight based on the number of votes, the lower the APs’ reference value. The abscissa indicates the trust threshold, and the ordinate indicates the number of APs votes. The higher the number of $AP_{5}$ votes, the higher the recognition rate of the algorithm for impaired APs. The lower the number of votes from other APs, the better the tolerance of the algorithm for environmental change. Therefore, the greater the difference between the numbers of votes obtained by $AP_{5}$ and other APs, the better the filtering performance of the algorithm.

As can be seen from Figure 8, the greater the value of Δ, the lower the recognition rate of impaired AP, but the ability to tolerate environmental interference is strong. When the value of Δ is small, the algorithm can absolutely identify impaired AP, but the algorithm’s ability to tolerate the environment is very low. When the environment changes slightly, it can affect the AP. It can be seen from Figure 8 that, when Δ=4, the algorithm not only has a better recognition rate of impaired APs, but also has some tolerance to environmental interference.

### 5.3. Effects of Different Impaired Levels

**Figure 9:**
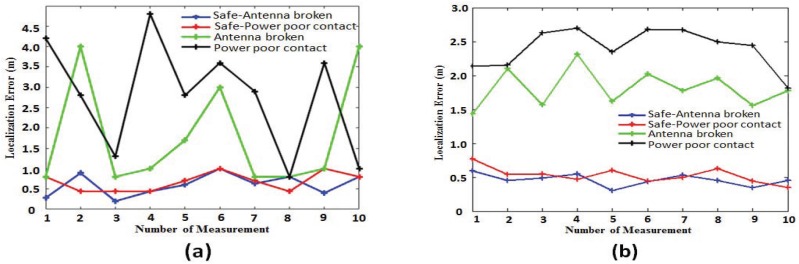
Error analysis of different impaired levels. (**a**) Multiple localization with the same location. (**b**) Error mean in multiple localization of different locations.

In the experiment, we placed an impaired AP for which the antenna of the AP was broken and an AP with poor power connection, respectively. First of all, we used the traditional fingerprint localization algorithm for localization and then used the safe fingerprint localization algorithm for localization. The localization results are shown in Figure 9.

Figure 9 is mainly used to observe the influence of different impaired levels on the location results. In order to analyze the impact of impaired AP on the localization results, we first conducted a single localization and analyzed the impact of impaired AP on the localization algorithm according to localization error, as shown in Figure 9a. Then, we selected multiple positions in the localization area for localization, where the localization error for each location is the average of multiple localization errors. Figure 9b shows the overall localization accuracy of the algorithm.

Figure 9a shows that 10 localization experiments were conducted at the same location. The black line indicates the localization result of the traditional fingerprint localization algorithm when the antenna of the AP is broken. The green line indicates the localization result of the traditional fingerprint localization algorithm for the AP with poor power connection. Since the RSSI of the impaired AP is unstable, the localization result in the same location is not stable, and the localization error largely changes. The red line and the blue line indicate the localization error of the algorithm proposed in this paper in the presence of different impaired levels in the environment. It can be clearly seen from the Figure 9a that the algorithm in this paper can guarantee better localization results when there are different impaired levels in the environment.

Figure 9b shows the average localization error for multiple runs at 10 different locations. It is mainly used to reflect the stability of the localization algorithm. In Figure 9b, the abscissa represents the different location, and the ordinate represents the average localization error of each location. For the AP with poor power connection, the black line represents the localization result of the traditional fingerprint localization algorithm, and the red line represents the localization result of the algorithm proposed in this paper. When the antenna of the AP is broken, the green and blue lines indicate the localization results of the traditional fingerprint localization algorithm and the proposed algorithm, respectively. As can be seen from Figure 9b, the localization algorithm proposed in this paper can effectively filter impaired APs and reduce the impact of impaired APs on the location result.

### 5.4. Effects of the Number of Impaired AP

**Figure 10:**
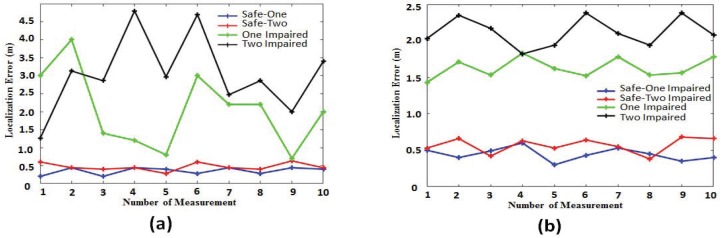
Error analysis of different impaired quantities. (**a**) Multiple localization with the same location. (**b**) Error mean in multiple localization of different locations.

It can be seen from Figure 9 that when an AP is impaired, the algorithm proposed in this paper can achieve a better filtering effect and eliminate the influence of impaired APs on the localization accuracy. However, there may be multiple impaired APs in the environment, so there is a need for experimental verification of the presence of multiple impaired APs. During the experiment, APs were first placed in the experimental environment, and one of the AP’s antennas was impaired, while the rest of the APs were normal. In this environment, the algorithm proposed in this paper and the localization algorithm without AP filtering are implemented. Then, in the experimental environment, two APs with impaired antennas were placed, and the remaining APs were normal. In this environment, the algorithm proposed in this paper and the location algorithm without the AP filter were implemented. The experimental results are shown in Figure 10. In Figure 10, the black and red lines indicate that there are two impaired APs in the environment. The green and blue lines indicate that there is an impaired AP in the environment. The black line and the green line represent the localization results of the traditional fingerprint localization algorithm that does not perform the AP security check. The red and blue lines represent the localization results of the proposed fingerprint localization algorithm.

Figure 10a shows that multiple localization are performed in the same location, and then, observe the impact of different numbers of impaired APs on the localization results. As can be seen from Figure 10a, when there is no AP security check, the more the number of impaired APs, the worse the localization result. When implementing the algorithm proposed in this paper, we can filter the impaired APs with good localization accuracy and maintain a good localization stability.

Figure 10b shows the average error for multiple localizations in multiple locations. As can be seen from Figure 10b, when the impaired APs in the localization environment increase, the localization error of the proposed algorithm increases, but the average localization error is controlled within 1 m. However, without the security checks of AP, the localization accuracy drops drastically as the number of compromised APs increases. Therefore, when the localization equipment is impaired in the environment, the algorithm proposed in this paper can well control the bad influence caused by the impaired AP and keep the localization system safe and reliable.

**Figure 11:**
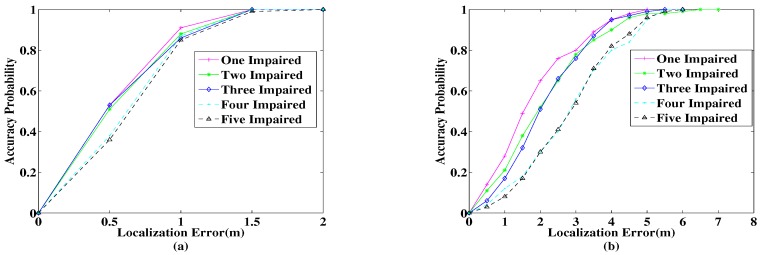
Error analysis of different numbers of impaired APs. (**a**) Error analysis of the proposed algorithm. (**b**) Error analysis of the traditional fingerprinting algorithm.

Due to the uncertainty of the degree of impaired APs in the localization environment, we need to observe the stability of the localization system as the number of impaired APs increases. In the experiment, we set different numbers of impaired APs and then make 100 locations in each case and calculate the probability distribution of their localization accuracy. Figure 11 shows the localization results for different numbers of impaired APs. The abscissa indicates the positioning error; the ordinate indicates the probability distribution of the error; and the lines of different colors indicate the number of impaired APs. Figure 11a shows the localization results of the proposed algorithm. Figure 11b shows the localization results of the traditional fingerprinting algorithm. Comparing Figure 11a,b, it can be clearly seen that whatever the number of impaired APs, as long as there are normal APs, the proposed algorithm can achieve a better localization effect. As can be seen from Figure 11, as the number of impaired APs increases, the localization error gradually increases. In the algorithm proposed in this paper, the more APs are impaired, the fewer APs are available for localization. Therefore, the localization errors change as the number of impaired APs increases. However, with the different numbers of APs used in localization, the RSSI can have different deviations. It can be seen from Figure 11 that the localization accuracy when the number of APs used for localization is large is not necessarily higher than the localization accuracy when the number of APs is small. The results shown in Figure 11 are not in strict accordance with the law of the more the number of impaired APs, the poorer the localization accuracy, and the localization results show some fluctuations. From the overall view of Figure 11, the localization accuracy will generally decrease as the number of available APs decreases.

### 5.5. Localization Accuracy

**Figure 12:**
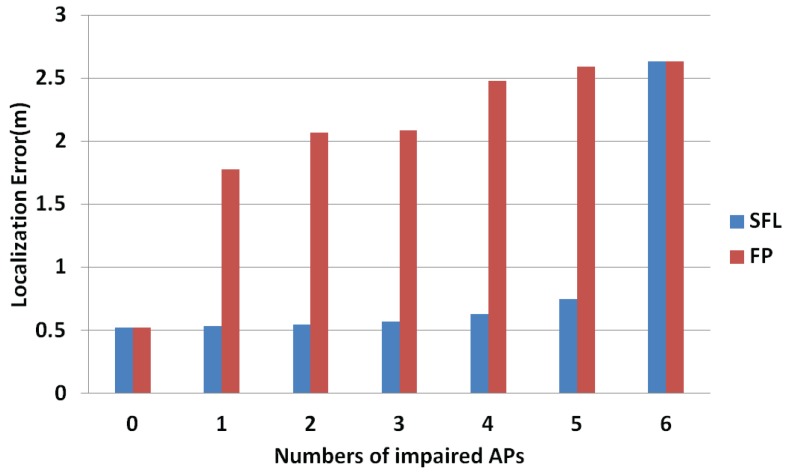
Algorithm positioning performance.

As the number of impaired APs increases, the stability of the localization system may change. In the actual scenario, the type and intensity of the disaster are random, such as different seismic levels and different intensity of explosion. In order to verify the localization performance of the system after different accidents, this paper carried out 100 localizations at different damage levels and used the average value of the 100 localization results as the localization performance index to evaluate the localization system at the scene of the disaster. As shown in Figure 12, the blue indicates the proposed algorithm, and the red indicates the traditional fingerprint localization algorithm. The abscissa indicates the number of impaired APs, and the ordinate indicates the average localization error. As can be seen from Figure 12, the localization performances of both algorithms decrease as the number of impaired APs increases. When there are impaired APs in the localization environment, the performance of the traditional fingerprinting localization algorithm drops sharply. However, the proposed algorithm can filter the impaired APs better and achieve better localization results. When all the APs are damaged, the algorithm proposed in this paper will have a sharp drop in performance. However, as long as there are normal APs, it can achieve a better localization effect. Therefore, when using the algorithm proposed in this paper, if it judges that all the APs have been impaired, the reference value of the localization result can be reduced during the rescue work.

**Figure 13:**
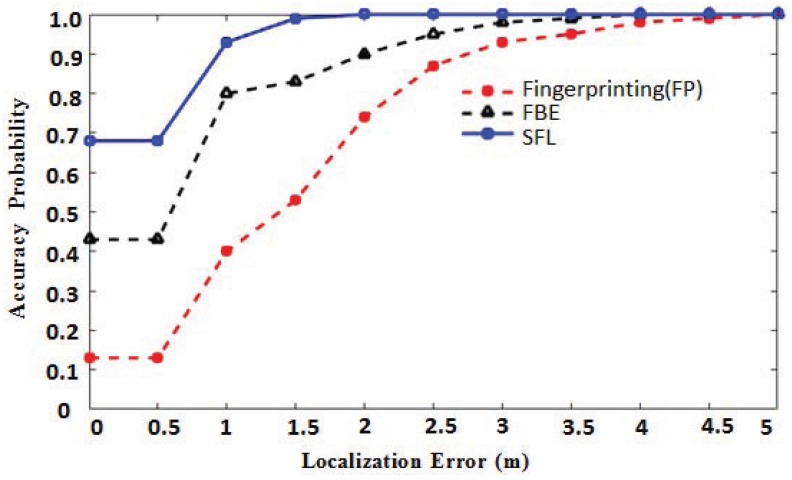
Localization accuracy analysis.

Figure 13 shows the superiority of the proposed algorithm. Figure 13 shows the localization accuracy obtained by performing different localization algorithms in the same experimental environment. In this experiment, we first placed an impaired AP in the location environment and then performed the proposed algorithm (SFL), the traditional fingerprinting localization algorithm (FL) and the AP filtering algorithm based on the exclusion method, respectively (FBE). The blue solid line represents the location algorithm proposed in this paper; the black dotted line represents the AP filtering algorithm based on the exclusion method; and the red dotted line represents the traditional fingerprint location algorithm. In Figure 13, the abscissa represents the localization error, and the ordinate represents the probability. In this experiment, 100 locations were located, and then, their localization errors were calculated.

Figure 10 shows only the positioning error in a small part of the positioning environment. The result cannot effectively reflect the positioning performance of the algorithm. In order to have a clear error analysis, we calculate the probability distribution of the localization error at intervals of 0.5 m, and the result is shown in Figure 13. There are many factors that affect RSSI in the localization environment, such as movement of people, metal interference, transfer of obstacles and collapse of buildings. The algorithm proposed in this paper can identify the APs whose RSSI is greatly disturbed. However, if the interference level of RSSI is within the threshold Δ, the algorithm only slightly reduces the reference value of the APs according to the number of votes and cannot completely eliminate APs that have been slightly disturbed. Therefore, after multiple positioning, nearly 10% of the probability will make the localization error 1 m or more.

As shown in Figure 13, when there is an impaired AP in the localization environment, the traditional fingerprint localization algorithm has a poor localization effect, and only 30% of the localization error is within 1 m. The fingerprinting algorithm based on the exclusion method is better than the traditional fingerprinting algorithm, with the localization error greater than 70% within 1 m. The algorithm proposed in this paper generally can achieve a better localization effect, and the localization error can be controlled within 1 m. Therefore, the algorithm proposed in this paper has better robustness to the harsh localization environment.

## 6. Conclusion

We researched the instability of the RSSI and analyzed the factors that caused the RSSI instability. Over time, physical device performance may deteriorate, equipment may be impaired and the localization environment may change; these factors can cause large changes in the RSSI. In this article, we mainly analyzed the influence of the changing environment, impaired APs, broken APs and newly-introduced APs on the localization system. We found that the instability of the RSSI is mainly the problem of fingerprint matching in the online phase and the offline phase. When the RSSI is unstable or new APs are added and the fingerprint database is unchanged, the difference in RSSI between the online phase and the offline phase is larger, resulting in an increase in localization error. Therefore, the choice of stable and credible fingerprints can effectively improve the localization accuracy.

We analyzed the changing characteristics of RSSI when the environment was disturbed and the physical equipment was impaired. We found a significant difference between the standard deviation of RSSI, the size of RSSI change and the affected range of RSSI. In addition, when collecting RSSI, the location and direction of the collection point will affect the size of RSSI. We proposed a new fingerprint database update method. The method uses the fixed reference anchor node for fingerprint database updating, which can reduce the interference factor when collecting training data. We proposed the voting mechanism, which can well identify the impact of different factors on RSSI and then extract the trust factor of the fingerprint and locate it by using safe and trusted location fingerprints.

According to the experimental verification, the proposed algorithm can identify the impaired APs well. In further work, a variety of studies will be possible by detecting impaired APs; for example, calculating the safety index of the area by detecting the impaired frequency of APs and making the hazard warning through the impaired area of APs to avoid unnecessary disasters. In addition, APs deployed in this article adopt an irregularly-shaped deployment. In future work, we can discuss how to deploy APs reasonably so that we receive the most APs in the location area.

## Figures and Tables

**Table 1:** The symbols used in this paper.

Symbol	Description
*N*	Number of grids
Ψ	Sampling matrix
*s*	The value of RSSI
*B*	Fluctuation matrix
*b*	Standard deviation of RSSI
*V*	The number of votes
φ	Measurement matrix
*D*	The distance matrix between grids
*r*	Anchor node communication radius
δ	Localization error
*L*	Number of anchor nodes
*T*	Localization error threshold
Δ	Standard deviation threshold
*w*	The trust value of fingerprint
$w^{\prime }$	The trust value of reference point

**Table 2:** The sequence of RSSI.

Location	AP${}_{1}$	AP${}_{2}$	AP${}_{3}$	AP${}_{4}$	AP${}_{5}$	AP${}_{6}$
$\left(x_{1},y_{1}\right)$	−68	−57	−53	−41	−74	−68
$\left(x_{2},y_{2}\right)$	−73	−59	−54	−44	0	−62
$\left(x_{3},y_{3}\right)$	−63	−52	−47	−32	−69	−61
…	…	…	…	…	…	…
$\left(x_{n},y_{n}\right)$	−58	−41	−46	−46	−70	−61
$\left(x_{i},y_{i}\right)$	−70	−57	−50	−40	−0	−66
